# Gewinnung von Echtzeitdaten aus der medizinischen Versorgung zur Handlungssteuerung in Public Health

**DOI:** 10.1007/s00103-021-03300-5

**Published:** 2021-03-24

**Authors:** Linus Grabenhenrich, MPH, Madlen Schranz, Sonia Boender, Theresa Kocher, Janina Esins, Martina Fischer

**Affiliations:** 1 Abteilung für Methodenentwicklung und Forschungsinfrastruktur (MF), Fachgebiet Informations- und Forschungsdatenmanagement (MF 4), Robert Koch-Institut, Nordufer 20, 13353 Berlin, Deutschland; 2 Klinik für Dermatologie, Venerologie und Allergologie, Charité – Universitätsmedizin Berlin, Berlin, Deutschland; 3 Abteilung für Infektionsepidemiologie (Abt. 3), Fachgebiet Surveillance (FG 32), Robert Koch-Institut, Berlin, Deutschland; 4 Abteilung für Infektionsepidemiologie (Abt. 3), Fachgebiet Infektionsepidemiologische Fach-IT und Anwendungsentwicklung (FG 31), Robert Koch-Institut, Berlin, Deutschland

**Keywords:** Public Health Surveillance, Versorgungsforschung, COVID-19, Computersysteme, Automation, Epidemiologie, Public health surveillance, Health services research, COVID-19, Computer systems, Automation, Epidemiology

## Abstract

Echtzeitdaten aus der medizinischen Versorgung spielen für die Handlungsteuerung in Public Health eine entscheidende Rolle. Dies zeigt die COVID-19-Pandemie besonders deutlich: Viele Public-Health-Akteure sind auf die aktuellen Daten aus dem klinischen und Versorgungsgeschehen angewiesen, um wichtige Entscheidungen treffen und Empfehlungen geben zu können. Die Automatisierung der Verarbeitungs- und Kommunikationsprozesse ist essenziell, damit eine Kontinuität des Datenflusses gewährleistet wird und Ressourcen geschont werden. Bisher standen der Entwicklung digital automatisierter Echtzeitsysteme mit wissenschaftlichem Qualitätsanspruch verschiedene technische, fachliche und organisatorische Herausforderungen im Weg. Die COVID-19-Pandemie dient seit ihrem Beginn Anfang 2020 als Motor für zukunftsfähige Systementwicklungen.

In diesem Beitrag wird zunächst beschrieben, wie ein Echtzeitdatensystem aufgebaut sein muss, damit eine automatisierte Datenverarbeitung möglich ist. Wichtige Aspekte bei der Zusammenführung der Daten, ihrer Aufbereitung, Bereitstellung und Kommunikation werden dargestellt. Als Beispiel dient ein System, das Routinedaten aus Notaufnahmen in Echtzeit verarbeitet und diese Public-Health-Akteuren bereitstellt. Es setzt sich zusammen aus dem Notaufnahmeregister des Aktionsbündnisses für Informations- und Kommunikationstechnologie in Intensiv- und Notfallmedizin (AKTIN) der Universität Magdeburg und der RWTH Aachen und dem Surveillance Monitor (SUMO) des Robert Koch-Instituts.

Die Entwicklung zukunftsfähiger Echtzeitsysteme zur Verarbeitung von Forschungsdaten aus der medizinischen Versorgung kann nur durch die Zusammenarbeit verschiedenster Akteure gelingen. Eine wichtige Grundlage für den langfristigen Erfolg ist die Entwicklung eines gesetzlichen Rahmens.

## Abstract (en)

Real-time data from medical care settings play an increasing role in guiding public health action. The COVID-19 pandemic is a good example; public health decisions depend on current data from the various clinical care settings. The automated processing and communication of health-related data is essential to ensure continuity of reporting and safe resources. So far, various technical, formal, and organizational challenges help back the development of digitally automated real-time systems with scientific quality standards. The COVID-19 pandemic pushed sustainable system developments since it began in early 2020.

This article describes how a real-time data system should be structured so that automated data processing is possible. Important aspects in the consolidation of the data and their preparation and communication are presented. The processes implemented for handling routine data from emergency departments in real time and making it available to public health actors is described. As an example, we present the cooperation between the emergency admission registry of the Aktionsbündnis für Informations- und Kommunikationstechnologie in Intensiv- und Notfallmedizin (AKTIN), the Universität Magdeburg, and the RWTH Aachen as well as the Surveillance Monitor (SUMO) hosted at the Robert Koch Institute.

The development of modern systems for processing research data in real-time from medical care settings can only succeed through the cooperation of a wide variety of actors. An important basis for long-term success is the development of a legal framework.

## Einleitung

Unzählige Einzelvorhaben und dauerhaft betriebene (Surveillance‑)Systeme sammeln weltweit Gesundheitsdaten für Public-Health-Akteure, als Grundlage evidenzgestützter Entscheidungen. Der Zeitraum zwischen Beobachtung (Datenerfassung) und Anwendung der Erkenntnisse kann dabei Wochen, Monate oder Jahre umfassen – für viele Fragstellungen der öffentlichen Gesundheit ist diese Verzögerung akzeptabel. Während der COVID-19-Pandemie wurde jedoch besonders deutlich, wie wichtig eine möglichst aktuelle und umfassende Datengrundlage zur zielgerichteten Handlungssteuerung ist. Die dafür notwendige Automatisierung der Verarbeitungs- und Kommunikationsprozesse ist dabei die Voraussetzung für Aktualität sowie ressourcenschonende Skalierbarkeit und Kontinuität. Bisher standen der Entwicklung digital automatisierter Echtzeitsysteme mit wissenschaftlichem Qualitätsanspruch vielerorts technische, fachliche und organisatorische Herausforderungen im Weg. Die COVID-19-Pandemie dient seit ihrem Beginn Anfang 2020 als Motor für die Entwicklung zukunftsfähiger Systeme.

Akute Gesundheitskrisen werden in Institutionen der primärmedizinischen Versorgung direkt erlebbar. Sie können wertvolle versorgungsnahe Daten liefern [1]. Gesellschaftliche Aspekte z. B. auf sozialer, individueller und wirtschaftlicher Ebene sind ebenfalls Teil der Evidenzgrundlage zur Entscheidungsfindung. Auch in diesen Bereichen gibt es Bestrebungen, möglichst aktuelle und umfassende Informationen zur Verfügung zu stellen, sie sind jedoch nicht Teil dieses Beitrags, der sich auf Datenquellen aus der medizinischen Versorgung beschränkt. Viele der im Folgenden besprochenen Aspekte können auf die Nutzung von Echtzeitdaten anderer Akteure des Gesundheitsbereichs übertragen werden, wie z. B. Gesundheitsämter, Diagnostiklabore und Einrichtungen mit Aufgaben in den Bereichen Vorsorge oder Rehabilitation.

Ein datenbasierter Einblick in das, was in der medizinischen Versorgung aktuell passiert, kann für sämtliche übergeordneten Ziele einer umfassenden Public Health Surveillance genutzt werden [2], wie etwa das frühe Erkennen neuer Gesundheitsgefahren. Die Daten ermöglichen ein kontinuierliches Monitoring der räumlich-zeitlichen Verteilung relevanter Gesundheitsphänomene und dienen der Analyse von Community-Interventionen oder bevölkerungsweiten Maßnahmen. Es sind immer mehr elektronische Versorgungsdaten verfügbar, die Informationen über die Gesundheit und das Inanspruchnahmeverhalten der Bevölkerung enthalten.

Dabei können 2 überlappende Informationsbereiche voneinander abgegrenzt werden: aktuelle klinische Daten und Daten zur aktuellen Versorgungssituation. Aus den in der Versorgungsroutine ohnehin erfassten Daten können Informationen zu Erkrankungen oder weiter gefassten individuellen patientenbezogenen Gesundheitsphänomenen abgeleitet werden, ggf. komplementiert durch Kenntnisse und Einschätzungen von Pfleger*innen und Ärzt*innen. Im Sinne einer syndromischen Surveillance können so Daten zu Häufigkeit, Schweregrad und Ausprägung relevanter Entitäten zusammengeführt werden und als Grundlage zur Public-Health-Handlungssteuerung genutzt werden [3]. Hierbei bezieht sich die Bezeichnung „syndromisch“ auf die vornehmliche Nutzung (i. d. R. wenig qualitätsgesicherter) klinischer Angaben, kann aber nach dem hier verwendeten umfassenderen Verständnis sämtliche Daten mit einbeziehen, die im jeweiligen Setting zur eindeutigen Definition abgrenzbarer (ggf. auch gewichteter) Fallkategorien verwendet werden (z. B. Angaben zur Diagnose, klinische Labordiagnostik). Im Rahmen der COVID-19-Pandemie konnten so z. B. Notaufnahmevorstellungen von Personen mit respiratorischen Leitsymptomen und intensivmedizinisch behandelte beatmete Patient*innen mit einer nachgewiesenen COVID-19-Erkrankung erfasst werden [4, 5].

Daten aus dem primärmedizinischen Kontext können auch verwendet werden, um Einblick in die aktuelle Versorgungssituation, Prozessabläufe sowie lokal verwendete und vorhandene Ressourcen zu erhalten. Üblicherweise arbeitet die Versorgungsforschung nicht mit Echtzeitdaten und zielt in ihrer Arbeitsweise auf langfristig zur Gestaltung der Versorgung nutzbaren Erkenntnisgewinn. Das schnelle Tempo der Veränderungen während der ersten Monate der COVID-19-Pandemie und die wiederkehrend beobachteten Versorgungsengpässe (z. B. in Norditalien, New York, in der zweiten Welle auch in Deutschland) zeigten jedoch, welche zentrale Rolle ein datenbasiertes Abbild der akuten Versorgungssituation für lokale und überregionale Public-Health-Entscheidungen einnimmt. Im Rahmen der COVID-19-Pandemie konnten so das Inanspruchnahmeverhalten von Notaufnahmen und die intensivmedizinischen Versorgungskapazitäten im zeitlichen Verlauf beschrieben werden – eine unmittelbare Entscheidungsgrundlage [4–6] z. B. für strategische Patient*innen-Verlegung regional und überregional.

Es sind vornehmlich staatliche Institutionen mit Public-Health-Auftrag, die als empfehlende bzw. entscheidende Gesundheitsakteure wahrgenommen werden: Gesundheitsämter, Landes- und Bundesbehörden, Gesundheitspolitik. Es gibt aber viele weitere Institutionen, die in ihrer Tätigkeit mit Empfehlungen und Entscheidungen zu Fragen der öffentlichen Gesundheit gefordert sind. Der Begriff „Public-Health-Handlungssteuerung“ bezieht hier *alle* strategischen und ggf. individuellen Fragen im Gesundheitsbereich mit ein, auch solche, die nicht direkt auf die individuelle Patientenversorgung abzielen. Aus einem datenbasierten Echtzeitabbild der aktuellen Versorgungslage profitieren so z. B. auch die für Präklinik zuständigen Steuerungsorgane, Management und Controlling von Krankenhäusern sowie nicht originär dem Gesundheitsbereich zugeschriebene, aber betroffene Akteure z. B. aus den Bereichen Bildung und Wirtschaft.

Der vorliegende Artikel beschreibt zunächst, wie ein Echtzeitsystem aufgebaut sein muss, damit eine automatisierte Datenverarbeitung aus dem jeweiligen Versorgungssetting bis hin zur Kommunikation und Anwendung als qualitätsgesicherte Grundlage für strategische Entscheidungen stattfinden kann. Exemplarisch wird ein bereits im ersten halben Jahr der COVID-19-Pandemie angepasstes System vorgestellt, das Routinedaten aus Notaufnahmen in Echtzeit verarbeitet und diese Public-Health-Akteuren bereitstellt (AKTIN-Notaufnahmeregister, SUMO-System). Es erfolgen Vergleiche mit einem Echtzeitsystem, dessen Aufbau vom Robert Koch-Institut (RKI) verantwortetet wird (DIVI-Intensivregister; [4–6]).

## Systemkomponenten und ihre Zusammenführung zur Bereitstellung der Daten

Auf konzeptueller Ebene werden nun die einzelnen (technischen, funktionalen) Bausteine eines Systems zur gemeinsamen Echtzeitverarbeitung von Daten aus den verteilt arbeitenden Institutionen der medizinischen Versorgung unabhängig von einer realen oder möglichen Implementierung generisch beschrieben. Zentraler Leitgedanke der hier skizzierten Perspektive auf eine solche Systemarchitektur ist, dass sämtliche Verarbeitungsschritte vollständig algorithmisch vordefiniert, technisch automatisiert und damit unabhängig von manuellem Zutun sind. Auch wenn im Kontext der Datennutzung (Public-Health-Handlungssteuerung) unter Echtzeit Größenordnungen von Stunden bis Tagen für die meisten Szenarien (Use Cases) ausreichend sind, ist ein ressourcenschonender Betrieb dauerhaft nur möglich, wenn die Verarbeitungsschritte ohne den Menschen durchgeführt werden. Analog zur sektorübergreifenden Translation von Erkenntnissen und Werkzeugen aus der Grundlagenforschung für klinische Entscheidungsprozesse und Maßnahmen (*From Bench to Bedside*) beschreiben wir hier den Transfer von Daten, Informationen und Erkenntnissen aus der medizinischen Versorgung für strategische Entscheidungsprozesse und Steuerung der öffentlichen Gesundheit (*From Care to Control*).

### Datenquelle

Die in der Versorgung ohnehin erfassten Daten über die Diagnose und Behandlung individueller Patient*innen sowie über Rahmenbedingungen der Versorgung sind, sofern digital verfügbar, die wichtigste Datenquelle. Die sekundäre Nachnutzung über die ursprünglichen Erhebungsgründe hinaus (z. B. zur Nachvollziehbarkeit der Behandlungsprozesse) erfordert jedoch, dass bereits bei der Erfassung möglichst einheitliche Sprachkonzepte verwendet werden. Dafür ist eine Anpassung lokal verwendeter Informationssysteme an übergeordnete Standards sinnvoll. So kann der spätere (technische, semantische) Übersetzungs- bzw. Zuordnungsaufwand (sog. Mapping) reduziert und den daraus resultierenden Fehlern vorgebeugt werden.

Werden für übergeordnete Entscheidungsprozesse jedoch Informationen benötigt, welche bisher nicht Teil der Dokumentationsroutine im jeweiligen Versorgungssetting waren, können ausgewählte Inhalte entweder zusätzlich primär erfasst werden (z. B. durch eigens dafür kurzfristig betriebene Erfassungstools) oder aus der hier beschriebenen Nutzungsperspektive als Ergänzung der Dokumentationsstandards vorgeschlagen werden. Um auf akut entstandene Erfassungsbedarfe im Rahmen einer sich rasch entwickelnden Gesundheitskrise zeitnah reagieren zu können, könnten in den Informationssystemen inhaltlich noch nicht definierte Datenfelder bereits technisch implementiert werden, welche zeitnah aktiviert würden. Alle diese Vorgehensweisen erfordern Mehraufwand seitens der datenliefernden versorgungstätigen Menschen und sollten daher auf das absolut notwendige Minimum reduziert werden, dem Leitgedanken der Ressourcenneutralität für versorgende Gesundheitsprofessionals folgend.

### Datenausleitung

Datensparsamkeit ist das wichtigste Kriterium für die Gestaltung der Verarbeitungsprozesse, welche Routinedaten aus der verarbeitungsverantwortlichen Versorgungsinstitution heraus der Nachnutzung zuführen. Auch wenn ein übergeordnetes Interesse an der Nutzung von z. B. besonders schützenswerten Individualdaten besteht, werden nur solche Inhalte zur Weiterverarbeitung außerhalb der Institution bereitgestellt, die für die Aufgaben der strategischen Steuerung im Bereich der öffentlichen Gesundheit absolut notwendig sind. Neben der Auswahl informativer Datenelemente und der lokalen Kategorisierung einzelner Elemente in die überhaupt notwendige Detailtiefe sollten Einzelpersonendaten so früh wie möglich im Verarbeitungsprozess auf das später auch zu nutzende, aggregierteste Niveau zusammengeführt werden. Insbesondere Informationen aus nicht vollständig standardisierbaren Erfassungen (wie z. B. Freitexte der Anamnese) müssen lokal in der verarbeitungsverantwortlichen Institution vorbereitet werden. Dort können zusätzlich mathematische Modellierungen angewendet und lediglich die Ergebnisse ausgeleitet werden.

Die hier beschriebenen Verarbeitungsprozesse können entweder direkt in den für die primäre Erfassung verwendeten Informationssystemen (z. B. Krankenausinformationssysteme/KIS) implementiert werden oder durch eigens dafür aufgesetzte, lokal ausgerollte Systeme realisiert werden, wie es in der Architektur des AKTIN-Notaufnahmeregisters (s. unten) beispielhaft umgesetzt ist [7]. Beide Ansätze machen eine Anpassung der Primärsysteme notwendig, was bei der großen Produktvielfalt eine Herausforderung für die Skalierbarkeit der in diesem Artikel beschriebenen Systeme ist. Der Einsatz eigens dafür entwickelter Zusatzsysteme wie in der AKTIN-Architektur reduziert als eleganter Lösungsweg diesen herstellerspezifischen Aufwand aber auf das Minimum der Schnittstellenentwicklung.

### Zusammenführung

Der direkte Weiterverarbeiter außerhalb des Versorgungssettings kann entweder eine treuhänderisch tätige Einrichtung (z. B. AKTIN) oder direkt die zentral für die weitere Datennutzung verantwortliche Institution sein (z. B. das RKI für das DIVI-Intensivregister). Spätestens bei der Zusammenführung von Sekundärdaten einer Art aus verschiedenen Quellen (z. B. mehreren Krankenhäusern) beginnen neben der Prüfung der Daten (Qualität, Inhalt, Plausibilität, Format etc.) auch die Anpassung und Bereinigung (Standardisierung). Für automatisierte Verarbeitungsprozesse ist dabei ein vordefiniertes, normatives Datenmodell wichtigste Grundlage. Für Primärdaten werden zwar bereits im Erfassungssystem bestimmte Prüfregeln angewandt, allerdings dienen diese oft nur der Qualitätssicherung mit Blick auf die primären Nutzungsziele und werden nicht im Hinblick auf die Vereinheitlichung über das lokal genutzte System hinaus gewählt. Daher müssen im zentralen Verarbeitungsprozess weitere Prüfregeln ergänzt werden.

Die Veränderung der erfassten Daten nach den Vorgaben des Datenmodells wird vor allem bei einmalig durchgeführten Forschungsvorhaben i. d. R. manuell durchgeführt. In einem Echtzeitsystem muss die manuelle Anpassung der Daten durch eine automatisierte Datentransformation ersetzt werden. Zur Gewährleistung der vollständigen Transparenz und Nachvollziehbarkeit werden die Veränderungsschritte dabei umfangreich dokumentiert. Der Leitgedanke für die Entwicklung einer komplett automatisierten Prüf- und Korrekturpipeline für qualitätsgesicherte und transparente Forschungsdaten ist daher, dass Auffälligkeiten und Anpassungen vollständig und nachvollziehbar dokumentiert werden. Eine solche Dokumentation der Verarbeitungsschritte (Logging) bietet auch die Möglichkeit, systematische Fehler zu identifizieren und in der Implementierung der vorherigen Verarbeitungsschritte zu korrigieren.

### Aufbereitung

Sobald die Daten im vorgegebenen Schema bereinigt vorliegen, können Informationen aus den Daten z. B. im Hinblick auf Surveillance-Fragestellungen extrahiert und Anwendungen für Akteure entwickelt werden. Da die hierbei verarbeiteten Daten i. d. R. für einen anderen Zweck erhoben wurden, ist die Operationalisierung ohne (oder mit nur bedingter) Möglichkeit zur Einflussnahme auf den primären Erfassungsinhalt eine besondere Herausforderung. Der Informationsgehalt einzelner Datenelemente für die zu untersuchenden Inhalte ist häufig nicht unmittelbar erkennbar. Im Anwendungsbereich der syndromischen Surveillance ist z. B. die auf Individualdaten aus der Versorgung basierende Falldefinition ein für einzelne Gesundheitsphänomene differenzierter Prozess und muss für die Nutzung in Echtzeitsystemen technisch implementiert werden (können). Um Gesundheitsdaten in Echtzeit für Forschung und Anwendung verwendbar und aussagekräftig zu machen (z. B. informative Aggregatindikatoren), müssen diese automatisiert mit geeigneten Bezugsgrößen in Relation gesetzt und mit Kontextdaten zusammengeführt werden (z. B. über die untersuchte Bevölkerung).

Erfordert das Anwendungsszenario eine Detektion bestimmter Verteilungsmuster, wie z. B. die Früherkennung von saisonalen oder regional veränderlichen Krankheitshäufigkeiten, müssen auch die sonst manuell begleiteten Beurteilungsschritte algorithmisch abgebildet und damit automatisiert werden [8].

### Bereitstellung und Kommunikation

Die so vorbereiteten Daten sollten Akteuren mit berechtigtem Nutzungsinteresse in dieser Form (ggf. sogar zusammen mit roheren, aber geprüften Vorstufen) im Sinne einer maximalen Datenwertschöpfung direkt zur Verfügung gestellt werden. Veränderliche Rahmenbedingungen für eine „FAIRe Datenverarbeitung“ [9] sollten dabei automatisiert angepasst werden (z. B. Anpassung von Verweisen, DOI-Vergabe).

Neben maschinenlesbaren vollständigen Datenformaten sollte ein modernes Forschungsdatenzentrum auch eine Umgebung zur manuellen Exploration und damit direkten Nutzung durch (Fach‑)Anwender*innen zur Verfügung stellen, z. B. in Form generisch vorbereiteter digitaler Visualisierungswerkzeuge (Dashboards, [10]). Ergänzt durch dauerhaft gültige Kontextinformationen und generische Interpretationshilfen entstehen daraus kontinuierlich aktualisierte, datenbasierte Berichtsformate [4, 11]. Für alle Formen der kontinuierlichen Bereitstellung von Daten und datenbasierten Berichten ist es notwendig, unter Berücksichtigung von Fragen des Datenschutzes und des Missbrauchspotenzials differenzierte Nutzergruppen getrennt zu adressieren.

Hier enden die Möglichkeiten einer vollständig automatisierten Datenverarbeitungskette. Die folgenden Schritte der Erkenntnisgewinnung und -anwendung bedürfen der manuellen Interpretation durch Expert*innen, sei es zunächst an zentraler Stelle (also direkt bei der für die Bereitstellung verantwortlichen Institution [12]), regional oder sektoral verteilt, oder direkt in der Verantwortung steuernd tätiger Akteure. Die Rückkopplung über die tatsächliche Nutzung sollte als Validierung zur konzeptuellen und inhaltlichen Weiterentwicklung solcher Systeme genutzt werden.

Die sonst übliche Bereitstellung von wissenschaftlich fundierten Daten, Informationen und Erkenntnissen folgt dem Konzept der einmaligen und unidirektionalen Kommunikation. Diese zentral in der wissenschaftlichen Arbeitsweise etablierte Kommunikationsform ist nicht für den Einsatz von echtzeitverarbeitenden Systemen ausgelegt.

## Implementierung für das Setting Notaufnahme

Beispielhaft wird im Folgenden das in der Pandemie weiterentwickelte System zur Bereitstellung von Daten aus dem medizinischen Versorgungssetting Notaufnahme für strategische Public-Health-Handlungssteuerung vorgestellt. Dieses Vorhaben verfolgt das Ziel eines vollständig automatisierten und damit kontinuierlichen und skalierbaren Betriebs und berücksichtigt sämtliche Aspekte der zuvor beschriebenen Verarbeitungskette.

Das Notaufnahmeregister des *Aktionsbündnisses für Informations- und Kommunikationstechnologie in Intensiv- und Notfallmedizin (AKTIN)*^1^ der Universität Magdeburg und der Rheinisch-Westfälischen Technischen Hochschule (RWTH) Aachen ist gemeinsam mit dem *Surveillance Monitor (SUMO)*^2^ ein System, das Routinedaten aus Notaufnahmen in Echtzeit verarbeitet, analysiert und für Public-Health-Akteure bereitstellt. Im April 2020 begann für SUMO die Pilotierungsphase, aus welcher es dann am RKI kontinuierlich gemeinsam von Epidemiolog*innen, Software-Entwickler*innen und Data Scientists weiterentwickelt wurde. Über die enge Kooperation mit dem AKTIN-Notaufnahmeregister und ergänzt durch Daten des ESEG-Projekts (Erkennung und Sicherung Epidemischer Gefahrenlagen) als Partner für Datenquellen und dezentrale IT-Komponenten wurden bereits während der Pandemie Routinedaten aus Notaufnahmen des gesamten Bundesgebiets zusammengeführt und nutzbar gemacht.

SUMO besteht aus einem Produktivsystem, Forschungs- und Entwicklungsbereichen und der Anwendungsebene. Das Produktivsystem gliedert sich in die im vorherigen Abschnitt generisch beschriebenen Systemkomponenten (Core Components).

### SUMO Core Components

Zunächst werden Notaufnahmedaten aus verschiedenen Primärsystemen zusammengeführt (Core Component 1, Source Data). Basis für diesen Prozess ist der normativ vorgegebenen Datenstandard *NoKeda*^3^ (Notaufnahme-Kerndatenmodell für Public-Health-Surveillance und Versorgungsforschung), mit welchem die Rohdaten überprüft und ggf. angepasst werden. Aus diesen so bereinigten Daten werden Hilfsvariablen und Gesundheitsindikatoren für die Definition von individuellen Fallkategorien abgeleitet (Core Component 2, Unroll Feature). Dieser kontinuierliche Datenstrom wird nun hinsichtlich Zeit, Raum und Eigenschaften (z. B. Syndrome) analysiert und ungewöhnliche Veränderungen (Signale) werden automatisch detektiert (Core Component 3, Manage Signals).

Die daraus erstellten Berichtsformate werden von Epidemiolog*innen vorbereitet und regelmäßig manuell inhaltlich ausgewertet. Wichtige Auffälligkeiten und für die Anwendung zentrale Kenngrößen können dabei zusammen mit weiteren Kontextinformationen bewertet werden und erlauben so eine auf die Nutzungsbedürfnisse fokussierte Ergebnispräsentation. Gemeinsam mit dem AKTIN-Notaufnahmeregister wurde ein automatisiert erstellter Notaufnahmesituationsreport entwickelt und über das RKI veröffentlicht [4].

### Dynamischer Systembetrieb

Die kontinuierliche Weiterentwicklung des SUMO-Produktivsystems während des Betriebs wird über Beiträge aus der Forschungsebene realisiert. Dort werden Methoden entwickelt, geprüft und als Teil der Software umgesetzt oder über Schnittstellen in bereits bestehende Systemkomponenten anpassend eingebracht. Das Produktivsystem bietet dabei eine Schnittstelle für jede Komponente. Damit kann das System dynamisch mit weiterentwickelten Methoden für neue Forschungsbedarfe zeitnah angepasst werden. Die Schnittstellen ermöglichen z. B. die Definition neuer Mappingregeln, neuer Syndrome/Falldefinitionen oder die Integration von neuen Signalerkennungsalgorithmen.

## Weiterentwicklung und Ausblick

Wir sind in Deutschland und international mit der Entwicklung, Erprobung und dem Betrieb automatisierter Datenverarbeitungssysteme in der Lage, daten- und damit evidenzbasiert Public-Health-Entscheidungen zu treffen, welche auch die aktuelle Lage der medizinischen Versorgung direkt berücksichtigen. In einem nächsten Schritt wird es darüber hinaus möglich sein, den Erfolg strategischer lokaler und überregionaler Maßnahmen datenbasiert zu untersuchen und einzuschätzen.

Die raschen Veränderungen im Verlauf der COVID-19-Pandemie haben den Bedarf von aktuellen Einsichten in zentrale medizinische Versorgungsbereiche gezeigt. Wesentliche Motivation für den kontinuierlichen Betrieb auch außerhalb von Krisen sind zum einen die Vorbereitung akut eintretender Ereignisse im Sinne eines technisch etablierten Zugangs zu Datenquellen (*Data Preparedness*) und zum anderen die Nutzung dieser Systeme als Forschungsplattform, z. B. für Fragen der Versorgungsforschung. In Echtzeit können diese Systeme auch außerhalb von Gesundheitskrisen datenbasierte Steuergrößen liefern und so differenzierte Abstimmung der Bedarfe und Angebote medizinischer Versorgung ermöglichen. Dabei können z. B. Aspekte der Patientensicherheit und der Qualitätssicherung über den Nachweis von Versorgungsstandards kontinuierlich beobachtet werden.

Aktuell gibt es keine Gesetzesgrundlage, die den Betrieb solcher Systeme regelt, welche aber vor allem für Fragen des Datenschutzes und der Finanzierung eine zentrale Rolle spielt. Auch Aspekte wie die Betriebsverantwortung und Beteiligung (Response, Coverage) können über die Entwicklung geeigneter rechtlicher Rahmenbedingungen gesteuert werden.

Auch wenn wir aufgrund pragmatisch gewachsener Zuständigkeiten, teilweise spärlicher Ressourcen und der sehr unterschiedlichen Interessen und Perspektiven beteiligter Akteure aktuell noch eine Entwicklung klar abgegrenzter Einzelsysteme sehen, gibt es generische Eigenschaften und Komponenten dieser Systeme, welche auf einer übergeordneten Ebene erlauben, über eine mittel- und langfristig zusammenführende und sich gegenseitig unterstützende Betriebskultur nachzudenken. Eine solche Entwicklung ist nur möglich, wenn sich die unterschiedlichen Akteure (aus der Versorgung, aus Institutionen mit Kenntnissen zu Forschungsdaten, aus Informationstechnologie und Epidemiologie sowie aus der lokalen und überregionalen öffentlichen Gesundheitspflege) über die gesamte Datenwertschöpfungskette hinweg zusammentun und gemeinsam an Lösungen für die Zukunft arbeiten.

## Footnotes

1 www.aktin.org.

2 https://www.rki.de/DE/Content/Institut/OrgEinheiten/Abt3/FG32/sumo/sumo.html.

3 https://art-decor.org/art-decor/decor-project--nokeda.
